# Forensic‐geology‐based magnetic analysis of beach sediments from the Shimokita Peninsula, Japan

**DOI:** 10.1111/1556-4029.15667

**Published:** 2024-11-14

**Authors:** Noriko Kawamura, Takuya Matsushita, Hiromi Itamiya, Ritsuko Sugita, Toshitsugu Yamazaki

**Affiliations:** ^1^ Japan Coast Guard Academy Kure Japan; ^2^ National Research Institute of Police Science Kashiwa Chiba Japan; ^3^ Atmosphere and Ocean Research Institute University of Tokyo Kashiwa Chiba Japan

**Keywords:** beach sediment, elemental mapping, forensic geology, inclusion, magnetic analysis, magnetic mineral

## Abstract

The occurrences of various illegal activities on beaches require effective geological and environmental investigation methods. Among these methods, the room‐temperature magnetic analysis of soils and sediments represents a nondestructive investigation method for various amounts, types, and grain sizes of magnetic minerals. Here, to verify the usefulness of magnetic analysis in forensic geology research, beach sediment samples from nine sites in the Shimokita Peninsula, Japan, were measured using magnetic analysis to determine the correlations between their concentration‐dependent magnetic parameters and actual regional characteristics. The results revealed that the values of various parameters, namely the low‐field magnetic susceptibility, anhysteretic remanent magnetization, and isothermal remanent magnetization (IRM), were relatively higher at sites near Ti and Fe sedimentary ore deposits. Further, thermomagnetometry results revealed that magnetite was the main magnetic carrier of the sediments. Moreover, pyrrhotite was detected around Ti–Fe mine sites. Furthermore, the results of the investigated parameters reflected the regional characteristics of the amount of magnetic minerals in the beach sediments. Low‐temperature IRM curves and the magnetic grain size parameter also displayed sample‐site‐reflective characteristics. Thus, we believe that magnetic analysis represents an effective method for estimating the provenance of beach sediments in forensic geology research.

**Highlights**

- Magnetic mineral assemblages and the grain size of beach sediments exhibit regional characteristics.
- Concentration‐dependent magnetic parameters are useful for sediment characterization and distinction.
- Magnetic parameters correlate with the count number of magnetic minerals.

## 1 INTRODUCTION

Sediment and soil samples collected from clothing, footwear, cadavers, and weapons are often employed as exhibits in criminal investigations. During such investigations, these samples are generally subjected to color, grain size, and elemental analyses [1]. Generally, mineralogical analysis methods, such as X‐ray diffraction (XRD) and energy‐dispersive X‐ray spectroscopy (EDX), require the preprocessing of samples, resulting in the modification of large amounts of materials, as well as changes in the chemical composition. Thus, if sample preservation is required, nondestructive analytical methods are desirable [1].

Nondestructive techniques are highly valued ideal tools for forensic examinations. The in‐field magnetic susceptibility of soils and sediments demonstrates a nondestructive method [1, 2]. Although low‐temperature and room‐temperature laboratory‐scale magnetic analyses consume soil and sediment samples, they do not cause chemical changes [3, 4, 5, 6, 7]. Further, the types, amounts, and particle sizes of magnetic minerals in samples revealed by magnetic analysis can be widely used in estimating their provenance [3, 4, 5, 6, 7].

Laboratory‐scale magnetic susceptibility (*χ*) is one of the most useful magnetic parameters in various methods. It is a concentration‐dependent rock magnetic parameter, reflecting the amount of magnetic minerals in samples. The *χ* value of soil and sediment samples represents a successful discrimination index and can offer valuable information about the provenance area in forensics [3, 4, 5, 6, 7, 8, 9, 10, 11]. In this regard, Guedes et al. [8] identified the *χ* of soil along a coastal area as an index for estimating the intrusion routes of illegal entry.

In addition to *χ*, forensic‐based magnetic analysis proceeds through anhysteretic remanent magnetization (ARM), which is a parameter of fine‐grained magnetic minerals, and thermomagnetometry. For example, Chen et al. [11] reported the *χ*‐ and ARM‐based investigation of the provenance of soils dumped on a highway. However, only a few studies have applied ARM to the estimation of the differences and regional characteristics of soils and sediments, necessitating the verification of its effectiveness in forensic geology. Therefore, in this study, we verified the applicability of magnetic analysis to forensic science using samples from Japan.

Illegal activities are sometimes conducted on beaches, and sediments from these beaches are used to examine exhibits recovered from suspects and tools. Various methods are used in this regard, and multiple principles and techniques are combined for such investigations [1]. For our verification, we selected beach‐sediment samples collected from nine sites in the Shimokita Peninsula of Aomori Prefecture, Japan (Table 1). In this respect, Itamiya et al. [12] notably reported the count of magnetic minerals, grain sizes, and surface microtextures of quartz found in an area to estimate the provenance and sedimentary process. As quartz is a common mineral in many rocks and sediments, the morphologies and surface microtextures of quartz particles from beach sediments in Japan can reflect the geological diversity, topography, precipitation, and artificial reclamation of an area in Japan. In addition to quartz, other minerals and/or rock fragments in an area may offer information for discrimination. Notably, the elemental compositions of the surfaces of beach sediments can be affected by pH changes caused by seawater (alkaline) or rainfall (acidic) [13]. Conversely, sedimentary‐particle inclusions, which are common in sediments and soils, are less susceptible to pH changes and thus offer relevant information about host rocks and their locations [13, 14].

**TABLE 1:** Sampling locations, date, and grain size of the beach sediments.

Sample ID	Site	Longitude (N)	Latitude (E)	Sampling date	Grade and class of grain‐size scales	Median diameters (Φ)
I‐1	The Pacific Ocean	41°05′00.46″	141°23′32.61″	August 3, 2014	Coarse sand	0.8
I‐2	The Pacific Ocean	40°58′19.27″	141°23’06.90″	August 3, 2014	Very fine sand	2.0
I‐3	The Pacific Ocean	40°49′47.22″	141°24’02.33″	August 3, 2014	Coarse sand	1.0
I‐4	The Pacific Ocean	40°44′13.98″	141°25′07.33″	August 3, 2014	Coarse sand	1.3
I‐5	The Pacific Ocean	40°42′37.29″	141°25′25.56″	August 3, 2014	Fine sand	2.1
I‐6	The Pacific Ocean	40°41′12.02″	141°25′52.74″	August 3, 2014	Very fine sand	2.4
I‐7	Mutsu Bay	41°11′48.05″	141°15′51.88″	August 4, 2014	Medium sand	1.2
I‐8	Mutsu Bay	41°06′04.81″	141°14′49.33″	August 4, 2014	Coarse sand	0.4
I‐9	Mutsu Bay	41°03′29.20″	141°14′32.23″	August 4, 2014	Very fine sand	2.0
	Ti‐Fe Mine 1	40°44′01.68″	141°24′42.19″			
	Ti‐Fe Mine 2	40°41′83.86″	141°26′16.12″			
	Ti‐Fe Mine 3	40°35′57.46″	141°27′59.94″			

*Note*: The grade and class of grain‐size scales and median diameters are modified after Itamiya et al. (2019) [12]. The locations of former Ti‐Fe mines are also presented [13, 14, 15].

On the basis of the foregoing considerations, this study was conducted to verify the applicability of magnetic analysis to the discrimination of various beach sediments using *χ*, ARM, thermomagnetometry, and elemental mapping of sample inclusions. Such measurements are summarized in Table 2, and the specific methods used are described below.

**TABLE 2:** The sample type and method.

Sample type	Preprocessing	Magnetic parameter measurements (χ, ARM, IRM, HIRM, and S_−0.3 T_)	Magnetic hysteresis curve and parameters (Hc, Ms., Mr., and Hcr)	Low temperature magnetometry (Verwey transition)	High temperature magnetometry (curie point)	FE‐SEM
Bulk sample	×	〇 Figures 2 and 5	〇 Figures 3, 4, and 8	〇 Figure 6	〇 Figures 6 and 8	×
Selected magnetic minerals	〇	×	×	×	×	〇 Figure 7
Quartz–feldspar particles	〇	×	〇 Figure 3	〇 Figure 6	×	〇

*Note*: A symbol “〇” indicates that the preprocessing and measurement were conducted at all sites. While a symbol “×” means that it was not carried out in this study. The obtained information by measurements is shown in the parentheses. Figure IDs are also indicated.

## 2 MATERIALS AND METHODS

### 2.1 Materials

**FIGURE 1:**
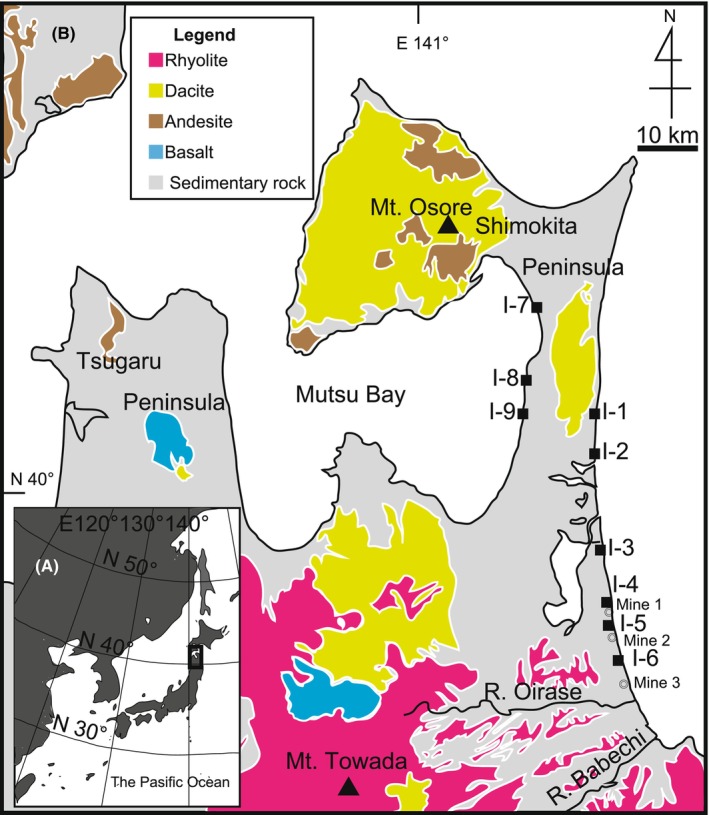
(A) Location map of Japan. The black square indicates the study area. (B) Geological map of Aomori Prefecture, with the sampling sites modified after Itamiya et al. (2019) [12]. Three samples were collected from Mutsu Bay beaches, whereas six samples were taken from the Pacific Ocean beach.

The study area covered the southern Shimokita Peninsula of Aomori Prefecture (northern Japan), which is in a volcanic front (Figure 1A). The northwestern part of the peninsula is home to two volcanos (Mt. Osorezan and Towada Volcano). The eruption and pyroclastic flows that birthed Mt. Osorezan (northwest of the peninsula) occurred approximately 480,000 years ago, and dacite and andesite are distributed around the crater (Figure 1B) [15, 16]. For the Towada Volcano (southern Aomori Prefecture), the last eruption occurred in 915 A.D., and volcanic ash was distributed around the prefecture [17]. Presently, rhyolite and basalt are distributed around the volcano (Figure 1B) [15].

The beach‐sediment samples investigated in this study were collected from nine sites in the Shimokita Peninsula in August 2014. As stated earlier, Itamiya et al. [12] partially analyzed these sediments. Sites I‐1, I‐2, I‐3, I‐4, I‐5, and I‐6 are on the eastern coast of the peninsula (the Pacific Ocean side). Sedimentary rocks, which formed during the Pleistocene and Holocene, are distributed around the sites. The sampling points were selected to capture different provenances. Thus, we believe that the study area is ideal for basic forensic research on sediment discrimination. Sample I‐1 was collected from the beach, which was near the dacite distribution. Lagoons were located in the hinterland between Sites I‐2 and I‐4, indicating that volcano‐derived particles might have been deposited therein. Further, ilmenite (FeTiO_3_)‐rich Ti and Fe sedimentary ore deposits were found around Sites I‐4, I‐5, and I‐6 [15, 18]. Furthermore, Ti–Fe‐rich sand was commercially collected (Figure 1B).

Additionally, the western sites (I‐7, I‐8, and I‐9) with sedimentary rocks (from the Holocene) are located on the Mutsu Bay coast (Figure 1B). No rivers exist around I‐7; meanwhile, small rivers flow near I‐8 and I‐9. The three samples were investigated to estimate the influence of rivers on the sediment particles from Mt. Osorezan.

Bulk samples of the beach sediments were collected from the back beach, which were then stored and divided for this study (Table 1).

### 2.2 Methods

The sediments were not sieved, and their particle sizes were not adjusted. As the total magnetic intensity of the samples is reflected in the species and grain size of magnetic minerals [4, 5, 6, 7], magnetic minerals must be concentrated for some part of the analyses. Three types of samples, ranging in size from a few tens to a few hundreds of milligrams, were used for the analyses (bulk samples, selected magnetic mineral samples, and quartz–feldspar particles).

The bulk samples contained magnetic minerals and quartz–feldspar, as they were not pretreated by sieving and washing.

The magnetic mineral samples were selected from the bulk samples using a Sm–Co magnet. The magnet was enclosed in a plastic capsule and brought in proximity to the diffused bulk samples on a Petri dish. Rock fragments and minerals that were on the capsule were collected. Almost all of the collected particles were black. Thereafter, the elemental mappings of the selected magnetic minerals were performed.

Furthermore, to verify the usefulness of regional estimation using inclusions, quartz–feldspar particles were randomly picked from the bulk samples under a stereomicroscope at the Japan Coast Guard Academy (JCGA). Almost all the quartz–feldspar particles contained opaque minerals. Additionally, pink and yellow minerals, such as αFeOOH, were sometimes detected in the particles. Finally, the samples were prepared for subsequent magnetic analysis and elemental mapping (Table 2).

#### 2.2.1 Measurement of the magnetic parameters of the bulk samples

The magnetic parameters, namely *χ*, ARM, and isothermal remanent magnetization (IRM), were measured. *χ* is the magnetic response dependent on the concentration of the magnetic fraction. *χ* and IRM values are concentration‐dependent rock magnetic parameters. *χ* values are reflected by the amount of paramagnetic, diamagnetic, and magnetic minerals in the sample [4, 5, 6, 7]. Meanwhile, IRM values depend on the amount of magnetic minerals in the samples. ARM depends on the relative proportion of fine‐grained magnetic minerals, such as the stable single‐domain grains. These parameters are explained in Table 3. The intensity of magnetic response reflects the magnetic minerals present. XRD or EDX are also used to determine the relative proportion of magnetic minerals. The proportion of minerals estimated using magnetic analysis may be different from those obtained using XRD or EDX depending on the sample.

**TABLE 3:** The explanations of magnetic parameters [3, 4, 5, 6, 7].

Parameter name	Abbreviation	Explanation
Magnetic susceptibility	χ	Magnetic response that is dependent on concentration of the magnetic fraction. A concentration‐dependent rock magnetic parameter. The amount of magnetic minerals.
Anhysteretic remanent magnetization	ARM	The relative proportion of fine‐grained magnetic minerals, such as the stable single‐domain grains.
Isothermal remanent magnetization	IRM	A concentration‐dependent rock magnetic parameter. The amount of magnetic minerals.
Hard isothermal remanent magnetization	HIRM	The absolute concentration of high‐coercivity material, such as hematite (Fe_2_O_3_) and goethite (αFeOOH).
S ratio (S_−0.3 T_)		The relative proportion of the low coercivity magnetic minerals, such as magnetite (Fe_3_O_4_)

The bulk sediment samples enclosed in plastic cubic boxes were used to measure *χ*, ARM, and IRM at JCGA. Samples were prepared by packing 0.09 to 0.14 g of bulk material into 6.7 cm^3^ plastic cubic boxes (Natsuhara‐Giken Ltd., Japan) with petroleum jelly. This method was used because completely filling the cubic boxes with sample generated magnetic intensities too strong preventing determination of the parameters. The plastic box and petroleum were nonmagnetic; thus, they did not affect the magnetic properties of the samples. Itamiya et al. [12] detected over 500 sand particles with sizes of 0.1–1 mm in the bulk samples; these were classified into four groups: colored minerals, magnetic minerals, colorless minerals, and rock fragments and others. The percentage of magnetic minerals was adopted from Itamiya et al. [12].

Here, *χ*, representing the magnetic response dependent on the concentration of the magnetic fraction, was measured using a Bartington MS2 magnetic susceptibility meter. Meanwhile, the ARM of the samples was characterized using a steady direct current (DC) bias field of 0.1 T in a peak alternating field of 100 mT. Additionally, the samples were subjected to IRM (0.35–1.58 T) using a DC electromagnet (Keiko‐denki Ltd., Japan). Both parameters (ARM and IRM) were measured using a spinner magnetometer (Natsuhara‐GikenSMM‐85). These measurements were performed in a few minutes and caused no chemical changes. Furthermore, the ARM/IRM ratio indicated the relative proportion of fine‐grained magnetic minerals in the sample, such as stable single‐domain grains (25–80 nm) [3, 4, 5, 6]. Moreover, hard IRM (HIRM) and S_−0.3 T_ were calculated, following the method of Bloemendal et al. [3]:

(1) $$S_{-0.3\;T}=\left(1-{\mathrm{IRM}}_{-0.3\;T}/{\mathrm{IRM}}_{1.58\;T}\right)/2,$$

(2) $$\text{HIRM}=\left({\mathrm{IRM}}_{1.58\;T}+{\mathrm{IRM}}_{-0.3\;T}\right)/2.$$

In Equation (1), S_−0.3 T_ reflects the relative proportion of low‐coercivity magnetic minerals, such as magnetite (Fe_3_O_4_), in a sample. A high S_−0.3 T_ value indicates a relatively high Fe_3_O_4_ proportion [3, 4, 5, 6, 7]. Meanwhile, HIRM represents the absolute concentration of high‐coercivity materials. A high HIRM indicates a sample rich in high‐coercivity minerals, such as hematite (αFe_2_O_3_) and goethite (αFeOOH).

#### 2.2.2 Measurement of the hysteresis loops of the bulk samples and quartz–feldspar particles

To estimate the grain size distribution of magnetic minerals, hysteresis loop measurements and DC demagnetization (DCD) of a saturation remanent magnetization (M_r_) were performed on 10–30 mg bulk samples and quartz–feldspar particles using an alternating gradient magnetometer (Model 2900–02, Princeton Measurements Corporation) at the Atmosphere and Ocean Research Institute, The University of Tokyo. The maximum applied field was 1.0 T, and the field increment was 2 mT during the hysteresis and DCD measurements. Further, the coercivity (*H*_c_), saturation magnetization (*M*_s_), and *M*_r_ were determined from the hysteresis measurements, and the *H*_c_ of the remanence (*H*_cr_) was determined from the DCD results. Thereafter, the values of *H*_c_, *M*_s_, *M*_r_, and *H*_cr_ were used for the Day plot to estimate the magnetic grain size [19].

#### 2.2.3 Thermomagnetometry

We performed thermomagnetometry to identify magnetic minerals (the presence of magnetite, hematite, and/or goethite) in the bulk samples and quartz–feldspar particles. Low‐temperature magnetometry was performed using a Quantum Designs magnetic property measurement system (MPMS‐XL5) at the Marine Core Research Institute, Kochi University (MaCRI). For the experiment, the samples (~50 mg) were packed in a capsule, after which they were imparted with IRM at 5 K in a 2.5‐T field. Subsequently, the intensities of the samples were measured from 5 K to 300 K in a zero‐field at 2 K intervals. Afterward, first‐derivative values (*dIRM*/*dT*) were calculated. The kind of magnetic minerals in the samples were estimated according to the low‐temperature magnetic transitions.

Further, high‐temperature magnetometry was performed in a vacuum. The bulk samples (~5 mg) were packed in a small quartz cup (with a diameter and height of 5 and 10 mm, respectively). A magnetic field (0.3 T) was applied to the cup, and the induced magnetization (*J*_s_) was measured from 100°C to 700°C using a magnetic balance (model NMB‐89; Natsuhara‐Giken, Ltd.) at MaCRI. The maximum value of *J*_s_ was described as *J*_max_, and normalized values (*J*_s_ */J*_max_) were calculated. Thus, the kind of magnetic minerals in the samples was determined on the basis of the Curie points, which were estimated using the difference in *J*_s_ with the temperature and the difference that reached the minimum values. The detected magnetic minerals are summarized in Table 4.

**TABLE 4:** The summary of magnetic analysis.

Sample ID	Site	S_−0.3 T_ of bulk sample	Thermo magnetometry				
			Bulk sample			Quartz–feldspar particles	
			High temperature	Low temperature		Low temperature	
			Curie point (°C)	Magnetite (120 K)	Pyrrhotite (30–34 K)	IRM decreases from 5 K to 30 K	Pyrrhotite (30–34 K)
I‐1	The Pacific Ocean	0.624	572	×	×	△	×
I‐2	The Pacific Ocean	0.643	562	×	×	〇	×
I‐3	The Pacific Ocean	0.648	561	×	×	〇	×
I‐4	The Pacific Ocean	0.647	582	×	〇	〇	×
I‐5	The Pacific Ocean	0.696	568	×	〇	△	〇
I‐6	The Pacific Ocean	0.707	562	×	〇	〇	×
I‐7	Mutsu Bay	0.669	561	△	×	△	〇
I‐8	Mutsu Bay	0.631	569	×	×	△	△
I‐9	Mutsu Bay	0.690	594	×	×	△	×

*Note*: “〇” indicates that the appearance of element is noticeable, “△” means that it is recognized but unclear, and “×” shows that it is not detected.

#### 2.2.4 Elemental mapping of the selected magnetic minerals

Next, to detect and observe the distribution of the selected magnetic minerals in rock fragments and to confirm the consistency of the thermomagnetometry results, the minerals were embedded in an epoxy resin (Struers Ltd. EpoFix Kit) and polished for elemental analysis. After an ultrasonic cleaning process, all samples were coated with Pt in a vacuum (because the field‐emission scanning electron microscopy [FE‐SEM] of JCGA was focused on metallic material analysis, there was no carbon evaporation equipment). The characteristic X‐ray peaks of S and Pt overlapped. However, elemental mapping was performed to investigate the location of S in the selected magnetic minerals rather than for quantitative determination. Thus, we believe that there would be no issues. The samples were observed using FE‐SEM (JEOL Ltd., JSM‐IT800HL). Further, qualitative elemental analysis was performed using an energy‐dispersive spectrometer (JED‐2300, JEOL Ltd.) attached to JSM‐IT800HL (SEM–EDX) at JCGA. The accelerating voltage was set to 15 kV in a vacuum, and the working distance was 10 mm. The elemental mapping and surface analysis were measured once. The image size deployed for the mapping was 1280 × 960 px. The elemental compositions of the minerals are summarized in Table 5.

**TABLE 5:** Elemental mapping of the selected magnetic minerals.

	Sample ID								
	The Pacific Ocean site						Mutsu Bay site		
Element	I‐1	I‐2	I‐3	I‐4	I‐5	I‐6	I‐7	I‐8	I‐9
Oxygen (O)	〇	〇	〇	〇	〇	〇	〇	〇	〇
Sodium (Na)	〇	〇	×	〇	×	×	〇	〇	〇
Magnesium (Mg)	〇	〇	〇	〇	〇	〇	〇	〇	〇
Aluminium (Al)	〇	〇	〇	〇	〇	〇	〇	〇	〇
Silicon (Si)	〇	〇	〇	〇	〇	〇	〇	〇	〇
Sulfur (S)	×	×	×	〇	〇	〇	×	×	×
Potassium (K)	×	〇	〇	×	×	×	〇	〇	〇
Calcium (Ca)	〇	〇	〇	〇	〇	〇	〇	〇	〇
Iron (Fe)	〇	〇	〇	〇	〇	〇	〇	〇	〇
Titanium (Ti)	×	×	×	〇	〇	〇	×	×	〇

*Note*: A symbol “〇” indicates that the appearance of element is detectable, while “×” shows that it is not detected.

## 3 RESULTS

Herein, we first explain the analysis results of the bulk samples, after which we discuss those of the selected magnetic minerals and quartz–feldspar particles.

### 3.1 Magnetic parameters of the bulk samples

**FIGURE 2:**
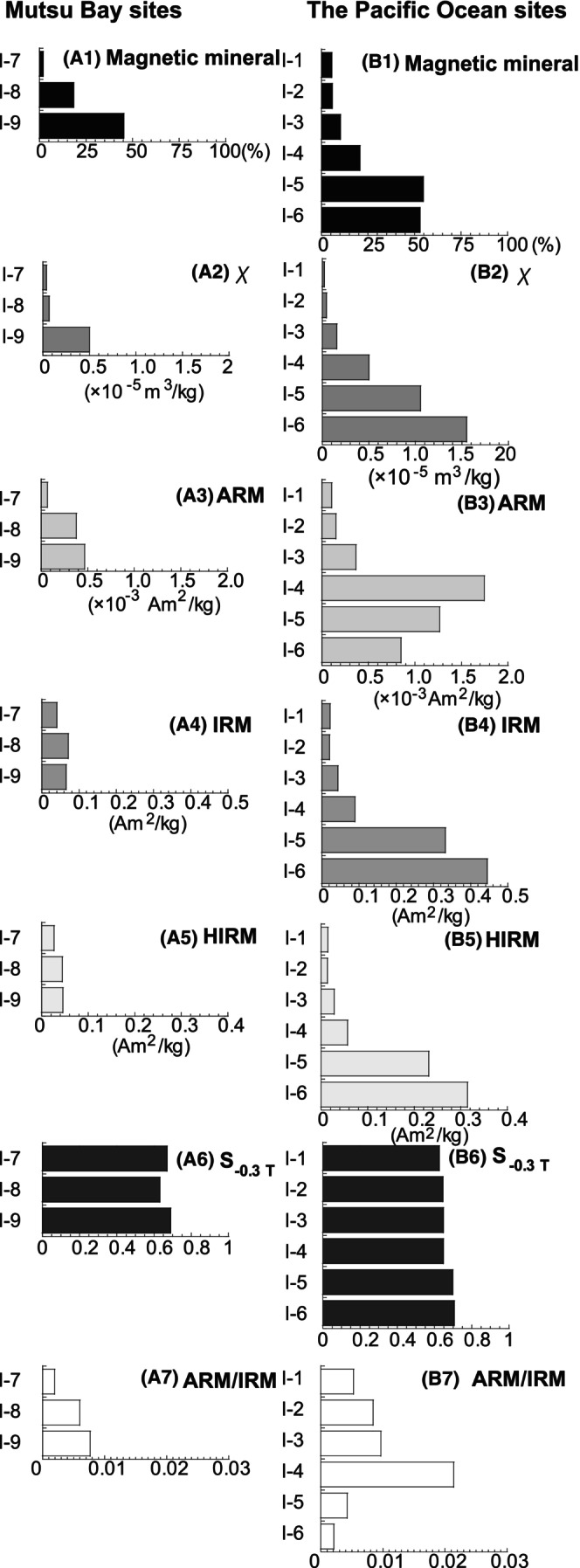
Results of the magnetic analyses of Mutsu Bay sites (A1–A7) and Pacific Ocean sites (B1–B7). The sampling locations are shown in Figure 1B. (A1 and B1) Magnetic mineral count in the bulk samples, modified after Itamiya et al. (2019) [12]. (A2 and B2) Regional variations of magnetic susceptibility; χ. (a3 and b3) Anhysteretic remanent magnetization (ARM). (A4 and B4) Isothermal remanent magnetization (IRM). (A5 and B5) Hard isothermal remanent magnetization (HIRM). (a6 and b6) S ratio (S_−0.3 T_). (A6 and B6) ARM/IRM, known as a magnetic grain size parameter.

Figure 2 and Table S1 show the magnetic parameters of the bulk samples. The concentration‐dependent parameter (i.e., χ) obtained a lower value at Sites I‐7 and I‐8 in Mutsu Bay, whereas the maximum value was observed at Site I‐9 (Figure 2A2,A4,A5). The lowest values of IRM and HIRM within Mutsu Bay were found at site I‐7. Further, the parameter values increased from Sites I‐1 to I‐3 at the Pacific Ocean sites (Figure 2B2,B4,B5). The average coastal currents were toward the southeast, as measured by the Japan Coast Guard in 2001–2008 [20]. Sites I‐4, I‐5, and I‐6, which were located near the Ti–Fe sedimentary mines where ferromagnetic minerals particularly likely gathered owing to the topography and water currents, also had relatively higher parameter values. Figure 2A1,B1 show the percentages of the magnetic minerals, which were modified following Itamiya et al. [12], displaying an increasing trend for *χ*. Meanwhile, Sites I‐4, I‐5, and I‐6 displayed high ARM values (Figure 2B3). The S_−0.3 T_ values of the sediment samples were very low, ranging from 0.62 to 0.70 and highlighting the presence of high‐coercivity magnetic minerals, such as hematite and goethite (Figure 2A6,B6) [3, 4, 5, 6, 7].

**FIGURE 3:**
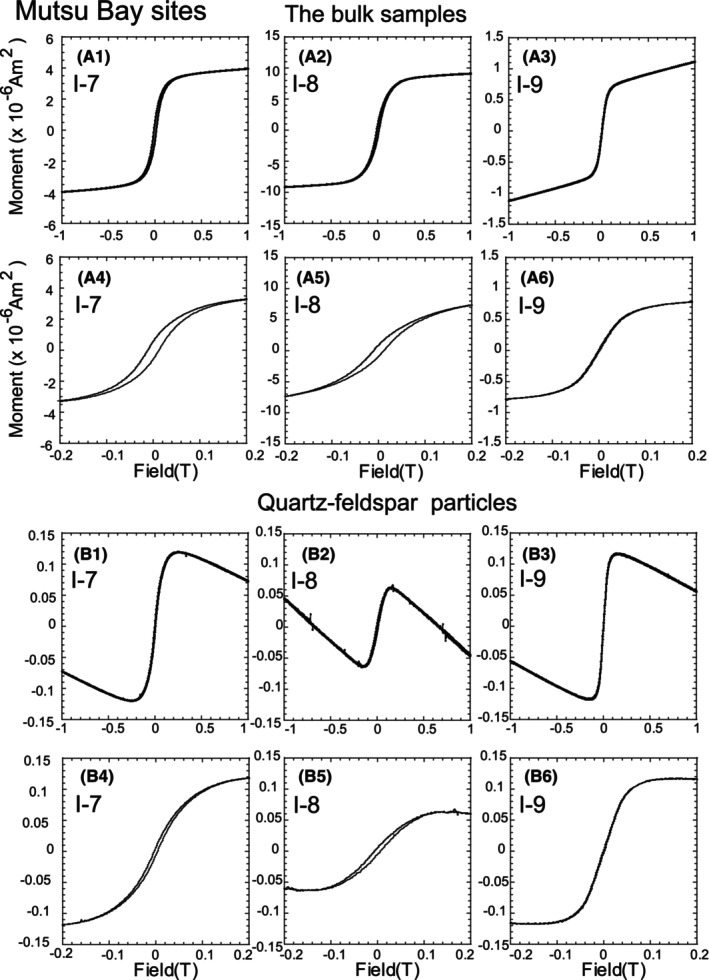
Hysteresis curves of samples collected from Mutsu Bay. (A1–A3) The curves of the bulk samples. The field range is from −1 to 1 T. (A4–A6) The field range is zoomed from −0.2 to 0.2 T. (B1–B3) Hysteresis curves of quartz–feldspar particles. (B4–B6) The field range is zoomed from −0.2 to 0.2 T.

**FIGURE 4:**
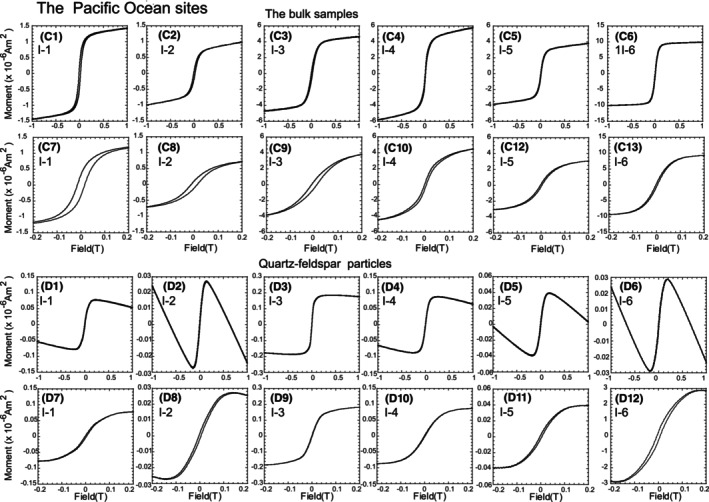
Curve of the Pacific Ocean site samples. (C1–C6) The field range is from −1 to 1 T. (C7–C13) The lower field range is zoomed from −0.2 to 0.2 T. (D1–D7) Hysteresis curves of quartz particles. (D7–D12) The field range is zoomed from −0.2 to 0.2 T.

The value of ARM/IRM is a magnetic grain size parameter if the magnetic carrier of the sample is mainly Fe_3_O_4_ [3, 4, 5, 6, 7]. The higher the ARM/IRM value, the greater the abundance of fine Fe_3_O_4_ in the samples (Figure 2A7,B7) [3, 4, 5, 6, 7]. A comparison of the ARM/IRM values with the grain size analysis results of Itamiya et al. [12] revealed that the grain distributions were not consistent with the magnetic grain size (Table 1). As subsequently explained herein, this observation indicated that the samples contained Fe_3_O_4_ and other magnetic minerals.

### 3.2 Magnetic hysteresis curves of the bulk samples

**FIGURE 5:**
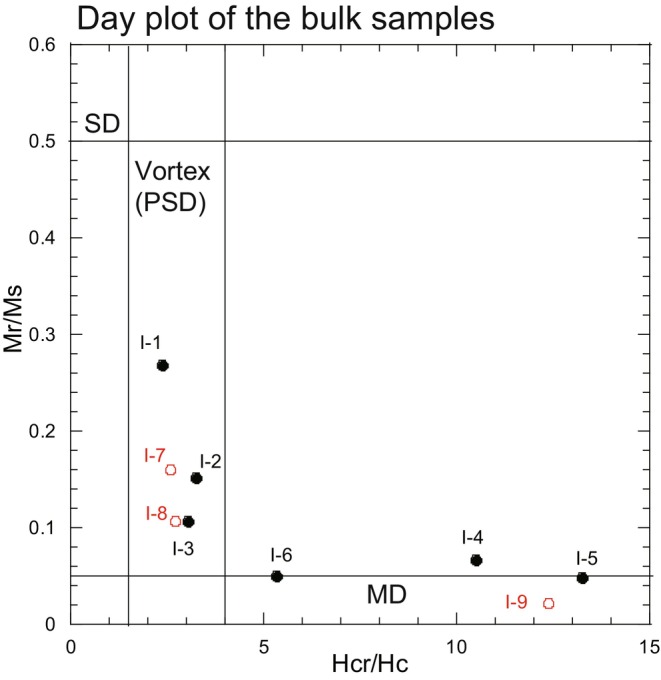
Magnetic grain size distributions of the bulk samples are shown on a Day plot (Day et al., 1977): Single‐domain (SD), vortex (the previous name was pseudo‐SD [PSD], but we used the notation together in this study), and multidomain (MD) [19, 20, 21, 22]. Red circles indicate that the beach sediment samples were collected from Mutsu Bay sites (I‐7, I‐8, and I‐9). The Pacific Ocean sites (I‐1, I‐2, I‐3, I‐4, I‐5, and I‐6) are shown as black circles on the plot.

The magnetic hysteresis curves of the bulk samples reflected ferrimagnetism (Figures 3A1–A6 and 4C1–C13) [3, 4, 5, 6]. We obtained *M*_r_, *M*_s_, *H*_cr_, and *H*_c_ data and calculated *M*_r_/*M*_s_ and *H*_cr_/*H*_c_ ratios based on these curves. These ratios were magnetic grain‐size‐dependent parameters and were used for the Day plot (Figure 5) [19]. A single domain (SD) indicated the possibility of capturing the magnetic field of a single particle in one magnetic domain, and the estimated size of Fe_3_O_4_ was ~0.03–0.15 μm [3, 4, 5, 6]. The vortex region (formerly known as a pseudo‐SD) exhibited a size of ~0.15–10 μm [3, 4, 5, 6, 20, 21]. The Fe_3_O_4_ grain size above ~10 μm was estimated using a multidomain (MD) [3, 4, 5, 6]. The samples from Sites I‐7 and I‐8 were located in the vortex region, whereas those from I‐9 were located in the MD region (Figure 5). In the Pacific Ocean sites, the northern‐site samples (I‐1, I‐2, and I‐3) corresponded to the vortex region. The southern‐site samples (I‐5 and I‐6) were plotted in the MD region, whereas the bulk sample from I‐4 was around MD on the Day plot [19]. These results indicated the feasibility of characterizing and discriminating magnetic grain sizes by sampling site.

### 3.3 Magnetic hysteresis curves of the quartz–feldspar particles

The hysteresis curves of the quartz–feldspar particles reflected diamagnetism, indicating that an applied magnetic field generated an induced magnetic field in them in the opposite direction [3, 4, 5, 6]. In other words, the magnetic field increased with decreasing moment. Such a phenomenon was observed in the hysteresis curves from −0.15 to −1 T and 0.15 to 1 T (Figures 3B1–B3 and 4D1–D6, respectively). However, the samples in this study displayed ferrimagnetism in the low magnetic field, from −0.2 to 0.2 T (Figures 3B4–B6 and 4D7–d12). Our results indicated that the quartz–feldspar particles comprised magnetic minerals as inclusions [13, 14, 23].

### 3.4 Magnetic mineral assemblages

**FIGURE 6:**
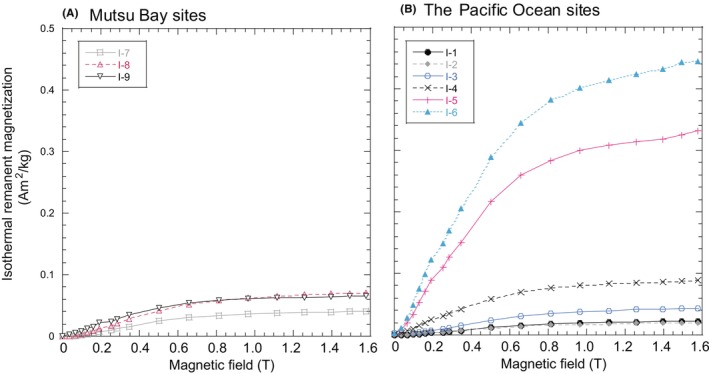
Isothermal remanent magnetization curves of the bulk sediment samples (A) collected from Mutsu Bay sites and (B1) for samples taken from the Pacific Ocean sites.

First, we estimated magnetic mineral assemblages in the bulk samples based on S_−0.3 T_ and IRM curves (Figures 2A6,B6, and 6 and Table 4). The S_−0.3 T_ results indicated that the magnetic mineral assemblages differed slightly with the sites, reflecting regional characteristics. Then, the IRM curves of the cubes were obtained to estimate detailed mineral assemblages (Figure 6A,B). For example, slight increases in IRM from 1.4 to 1.6 T were observed in samples from Sites I‐5 and I‐6 but were absent from other samples. This indicated the presence of high‐coercivity magnetic minerals in the bulk samples at Sites I‐5 and I‐6 (Figure 6B) [3, 4, 5, 6, 7].

**FIGURE 7:**
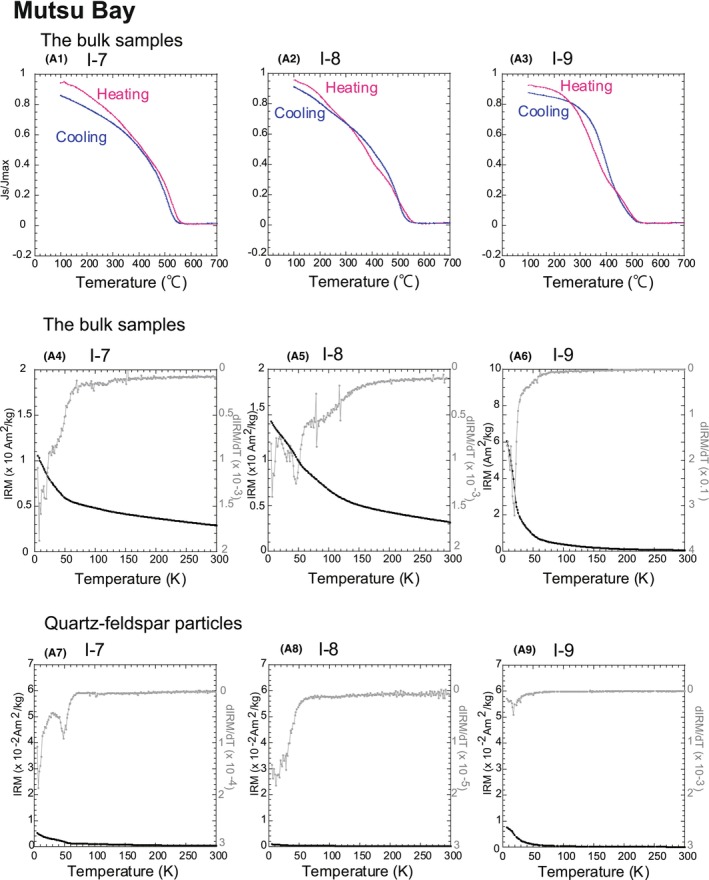
Thermal demagnetizations of the bulk samples and quartz–feldspar particles. The results for the Mutsu Bay samples are shown as (A). (A1–A3) Magnetization vs. temperature curves of Mutsu Bay samples. (A4–A6) Thermal demagnetization curves of the bulk samples for a low‐temperature IRM imparted at 5 K with a 2.5‐T DC field. (A7–A9) Thermal demagnetization curves of quartz particles. Solid squares represent IRM intensity, whereas gray lines correspond to *dIRM/dT*.

**FIGURE 8:**
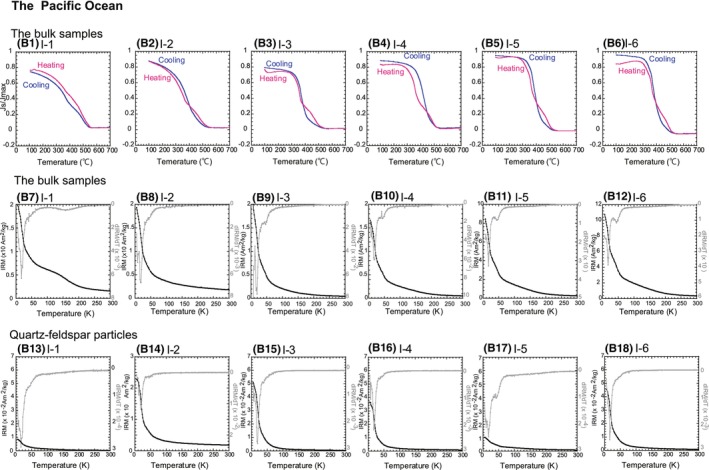
Results for the Pacific Ocean samples are shown as (B). (B1–B6) Magnetization versus temperature curves of the Pacific Ocean sites. The measurements were conducted in a vacuum, and magnetization values (*J*_s_) were normalized using the maximum value of magnetic susceptibility (*J*_max_). The pink curves indicate the values during heating, whereas the blue curves show cooling curves. (B7–B12) Thermal demagnetization curves of the bulk samples for a low‐temperature IRM imparted at 5 K with a 2.5‐T DC field. (B13–B18) Thermal demagnetization curves of quartz particles. Solid squares represent IRM intensity, whereas gray lines correspond to *dIRM/dT*.

Next, magnetic mineral identifications were performed using the results of high‐temperature magnetometry. The results for the bulk samples indicated a decrease from 300°C to 400°C (Figures 7A1–A3 and 8B1–B6) in a region known as the Curie point of pyrrhotite (Fe_7_S_8_, 320°C) or the thermal decomposition of maghemite and (titano)maghemite [4, 5, 6]. Assuming that magnetic mineral decomposition occurred at this point, Fe_3_O_4_ would be formed, and the *J*_s_/*J*_max_ values of the cooling curve must be much higher than that of the heating curve. However, we did not observe any increase in the *J*_s_/*J*_max_ values (Figures 7A1–A3 and 8B1–B6). The decrease from 560°C to 600°C was also observed in all the bulk samples (Figures 7A1–A3 and 8B1–B6). Notably, the Curie point of Fe_3_O_4_ was 580°C [4, 5, 6]. The temperature‐dependent difference in the *J*_s_ value was calculated [26], and the temperatures of the minimum *J*_s_ values from 560°C to 600°C are listed in Table 4. The Curie point for the Pacific Ocean sites ranged from 561°C to 582°C, whereas those for the Mutsu Bay sites ranged from 561°C to 594°C (slightly higher than those of the Pacific Ocean sites). Assuming that the magnetic minerals contained Ti, relatively low Curie points would have been observed [29].

**FIGURE 9:**
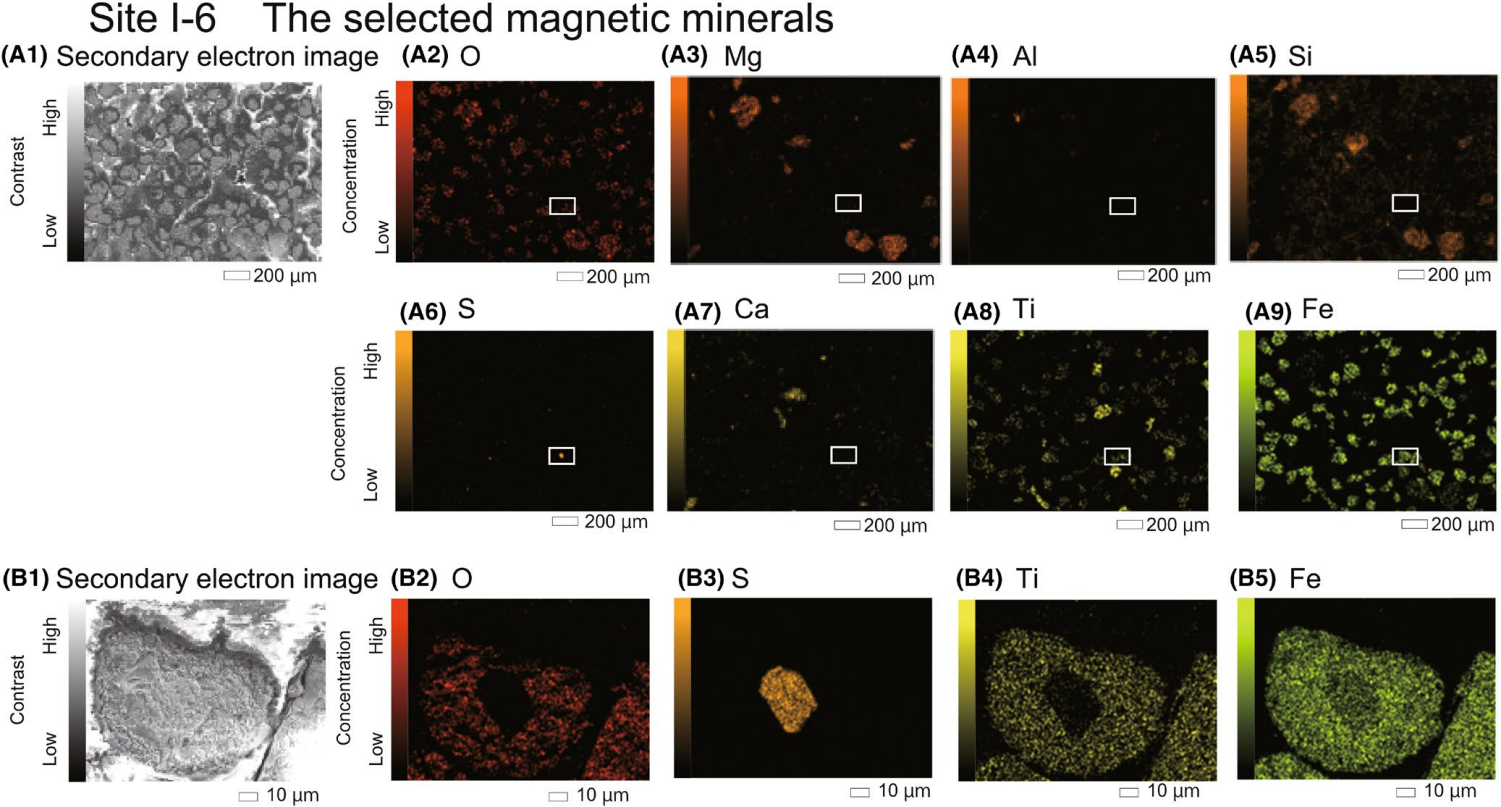
(a1) Secondary electron image of grains. (A2–A9) Element distributions in the selected magnetic minerals using a FE‐SEM in a Site I‐6 sample. The white square indicates the expanded area of Figures. 9B1–B5. (b1) Secondary electron image of a grain. (B2–B5) A pyrrhotite (Fe_7_S_8_) inclusion is recognized.

We also described the low‐temperature magnetometry of the bulk samples and identified magnetic carriers. Figures 7A4–A6 and 8B7–B12 show the results. The temperature‐dependent decreases in IRM were expressed as *dIRM/dT*; they reflected the mass and/or species of the magnetic mineral. The IRM warming curves of all the samples displayed unclear decreases at 120 K, representing the Verwey transition of Fe_3_O_4_ [25, 26, 27]. If the Verwey transition clearly appears, the presence of pure Fe_3_O_4_ is suggested. The decrease at 30 K–35 K in the samples from Sites I‐3, I‐4, I‐5, and I‐6 reflected the magnetic‐phase transition of Fe_7_S_8_ (Figure 8B10–B12) [29, 30].

Finally, we assessed the low‐temperature magnetometry of quartz–feldspar particles to estimate their magnetic inclusions. The low‐temperature magnetometry of the quartz–feldspar particles was obtained (Figures 7A7–A9 and 8B13–B18). The decrease in intensities from 5 K to 30 K was observed in almost all the particles, except for those from Site I‐8. As quartz exhibits diamagnetism, the intensities must be zero [4, 5, 6]. However, the curves obtained in this study were not consistent with this theory. Such a decrease reportedly reflects the presence of an iron hydroxide, such as αFeOOH and/or superparamagnetic grains [31, 32, 33]. The slight decrease around 40 K reflected the magnetic transition of Fe_7_S_8_ at Sites I‐5, I‐7, and I‐8, indicating that the quartz–feldspar particles contained magnetic minerals as inclusions (Figures 7A7,A8, and 8B17). The results are summarized in Table 4.

### 3.5 Elemental mappings of the selected magnetic minerals

Elemental mappings of the selected magnetic minerals were performed for all the samples. Table 5 summarizes the elements detected in the particles. As presented in the table, O, Mg, Al, Si, Ca, and Fe were detected in all samples (Table 5). However, the presence of Na, S, K, and Ti depended on the sites. Pt was difficult to detect as Kα and Lα peaks in the integrated spectrum could obscure the presence of other elements. For instance, Ti and S were detected in the samples from Sites I‐4, I‐5, and I‐6, which were located near the Ti–Fe mines (Table 1). Notably, Ti was not detected in samples from Sites I‐7 and I‐8, which were collected from the Mutsu Bay sites (Table 5), although it was found in Site I‐9.

Figure 9A2–A9 show elemental mapping images of the selected magnetic minerals in the Site I‐6 sample from the Pacific Ocean site. The magnification of a point where S was detected (Figure 9A6) revealed its spatial association with the presence of Fe (Figure 9B3,B5). The thermomagnetometry results revealed the presence of Fe_7_S_8_ in the Site I‐6 sample (Figure 8B6,B12 and Tables 4 and 5). Therefore, Fe_7_S_8_ was determined as an inclusion in the selected magnetic minerals. The thermomagnetometry results suggest the Curie point and the magnetic transition of Fe_7_S_8_, which were coincident with those of elemental mapping.

## 4 DISCUSSION

First, we verified the effectiveness of magnetic mineral assemblages as regional proxies. The decrease in *J*_s_/*J*_max_ values from 560°C to 600°C, which was near the Curie point of Fe_3_O_4_, was observed in all bulk samples (Figures 7A1–A3 and 6B1–B6), and a slight difference in the Curie point was observed per sites (Table 4). Anai et al. [34] compared the Curie points of the volcanic sediments and distinguished the particle origins. The site‐to‐site comparison of Curie points can be a valuable test method for discriminating the beach sediments in this study area (Table 4). Further, the Verwey transition could also be a regional proxy. The unclear Verwey transition of Fe_3_O_4_ at 120 K reflected the presence of Ti‐rich Fe_3_O_4_ [24]. Generally, Ti‐substituted (titano‐)magnetite exhibits low Curie points [24] and suppressed Verwey transitions [28]. Notably, Ti was detected through elemental analysis (Figure 9A8 and Table 5). Therefore, Fe_3_O_4_ accounted for the principal magnetic carrier of the bulk samples [4, 5, 6]. S, an Fe_7_S_8_ Curie point, and a magnetic transition were detected at Sites I‐4, I‐5, and I‐6, which were around the Ti–Fe mines (Figures 8B10–B12, 7A6,7B3 and Table 1). This further indicated that Fe_7_S_8_ was a magnetic carrier at those sites. Additionally, αFeOOH and FeTiO_3_ might be present in the samples, although the detections of these minerals were challenging in this study [4, 5, 6, 31, 32] because of the weak mass‐specific magnetic susceptibility of αFeOOH (1/500–1/1000 the intensity of Fe_3_O_4_) [5]. Moreover, FeTiO_3_ exhibited a significantly weaker intensity than that of Fe_3_O_4_ [5]. The low‐temperature magnetometry results indicated that the curves of the bulk samples were exclusive per site, especially the curves that were characteristic of Mutsu Bay (Figure 7A4–A6). The shapes of the curves were used to estimate the changes in the distribution of sediments in the coastal samples [35]. Thus, the comparison of the shapes of the curves might facilitate the estimation of regional characteristics. The decrease in the IRM of the quartz–feldspar particles from 5 K to 40 K suggested the presence of αFeOOH and/or Fe_7_S_8_. This might facilitate the discrimination of the samples (Figures 7A7–A9 and 8B13–B18 and Table 4).

Next, we verified the relevance of the amount of magnetic minerals in the beach‐sediment samples for their discrimination. We observed that the magnetic‐concentration‐dependent parameters, namely χ, IRM, and HIRM, increased from Site I‐1 to I‐3 in the Pacific Ocean samples (Figure 2B2,B4,B5). Such a trend was also observed in the parameters of the Mutsu Bay site samples (Figure 2A1). In this regard, Itamiya et al. [12] reported the magnetic mineral counts in the bulk sample, indicating that the ratio increased as one moved southward (Figure 2A1,B1) [12]. Furthermore, *χ*, IRM, and HIRM exhibited relatively high values at Sites I‐4, I‐5, and I‐6. Geological and mineral resource maps [16] reveal Ti–Fe sedimentary mines around Sites I‐4, I‐5, and I‐6 (Figure 1B). The magnetic mineral counts at Sites I‐4 and I‐5 were also higher than at the other sites, corresponding to the results of their magnetic parameters (Figure 2B1,B2,B4,B5). However, slight discrepancies between the magnetic mineral counts and magnetic parameters (*χ*, IRM, and HIRM) were observed at Site I‐6. The percentages of the magnetic mineral counts at Sites I‐6 and I‐5 were almost the same, although Site I‐6 exhibited the highest magnetic parameter values. The weakest magnetization minerals were counted at Site I‐5 [4, 5, 6]. Further, three sites displayed higher ARM values (Figure 2B3). Ti was detected in the selected magnetic minerals (Figure 9A8), whereas Fe_7_S_8_ was detected at the sites (Figure 8B10–B12 and Table 5). Fine‐grained Ti‐rich magnetic minerals and/or Fe_7_S_8_ might have contributed to the high ARM values. The distribution from the mines might have increased the *χ*, ARM, IRM, and HIRM values at Sites I‐4, I‐5, and I‐6. This indicated that the magnetic mineral abundance reflected regional characteristics.

Elemental mappings facilitated the discrimination of mineral species where magnetic analysis could not, such as in the presence of magnetization minerals exhibiting weak mass‐specific magnetic susceptibility (Table 5). The adoption of multiple analytical methods using different theorems is recommended in forensics. This recommendation is satisfied by combining magnetic and elemental analyses, as deployed in this study.

## 5 CONCLUSIONS

In this study, we subjected nine beach‐sediment samples from different sites in the Shimokita Peninsula (Aomori Prefecture, Japan) to magnetic analyses to validate the applicability of this analytical tool to forensic geology in Japan. Three of the samples were collected from Mutsu Bay beaches, whereas six were from a Pacific Ocean beach. Magnetic minerals were separated using a magnet, whereas quartz–feldspar particles were picked from the bulk sediment samples. The bulk samples, selected magnetic minerals, and quartz–feldspar particles were subjected to magnetic analyses, which revealed that the magnetic‐concentration‐dependent parameters almost corresponded to the results of the magnetic mineral counts. Relatively higher values of these parameters were obtained around the sites near the sedimentary Ti–Fe mines, indicating the abundance of magnetic minerals in the bulk samples. Further, the magnetic hysteresis parameters indicated the distribution of relatively fine‐grained magnetic minerals in the northern sites, whereas the magnetic grain sizes increased when moving southward. The thermomagnetometry and elemental mapping results revealed that Fe_3_O_4_ was the main magnetic carrier and that Fe_7_S_8_ was substantially detected around the Ti–Fe mine sites. The low‐temperature IRM curves were specific corresponding sites, and estimation methods were recommended for the inclusion amount in the minerals. Our results indicated that magnetic analysis is a valuable tool for discriminating magnetic‐mineral‐abundant beach sediments with varying mineral species proportions.

## FUNDING INFORMATION

This work was supported by JSPS KAKENHI Grant Number 20 K01153, 23 K17536, and the Nippon Foundation Fund for Maritime Safety and Security Studies. This study was performed under the cooperative research program of MaCRI (accept No. 20A006 and 20B005) with the support of JAMSTEC.

## CONFLICT OF INTEREST STATEMENT

The authors declare no conflicts of interest.

## Supporting information


**TABLE S1.** Sampling locations and the magnetic parameter values of the bulk beach sediments.
